# Optic atrophy, necrotizing anterior scleritis and keratitis presenting in association with Streptococcal Toxic Shock Syndrome: a case report

**DOI:** 10.1186/1752-1947-2-69

**Published:** 2008-02-29

**Authors:** Konstantinos I Papageorgiou, Alexander S Ioannidis, Petros S Andreou, Ajay J Sinha

**Affiliations:** 1 Department of Ophthalmology, Mid Essex NHS Trust, Court Road, Chelmsford, Essex CM1 7ET, UK

## Abstract

**Introduction:**

We report a case of optic atrophy, necrotizing anterior scleritis and keratitis presenting in a patient with Streptococcal Toxic Shock Syndrome.

**Case presentation:**

A 43-year-old woman developed streptococcal toxic shock syndrome secondary to septic arthritis of her right ankle. Streptococcus pyogenes (b-haemolyticus Group A) was isolated from blood cultures and joint aspirate. She was referred for ophthalmology review as her right eye became injected and the pupil had become unresponsive to light whilst she was in the Intensive Therapy Unit (ITU). The iris appeared atrophic and was mid-dilated with no direct or consensual response to light. Three zones of sub-epithelial opacification where noted in the cornea. There where extensive posterior synechiae. Indirect ophthalmoscopy showed a pale right disc. The vision was reduced to hand movements (HM). A diagnosis of optic atrophy was made secondary to post-streptococcal uveitis. She subsequently developed a necrotizing anterior scleritis.

**Conclusion:**

This case illustrates a previously unreported association of optic atrophy, necrotizing anterior scleritis and keratitis in a patient with post-streptococcal uveitis. This patient had developed Streptococcal Toxic Shock Syndrome secondary to septic arthritis. We recommend increased awareness of the potential risks of these patients developing severe ocular involvement.

## Introduction

Post-streptococcal uveitis is a rare complication of streptococcal infection.

We present a case of optic atrophy, necrotizing anterior scleritis and keratitis in a female patient with post-streptococcal uveitis who was admitted with Streptococcal Toxic Shock Syndrome.

## Case presentation

A 43-year-old female initially presented to her general practitioner with rigours and a red, swollen right ankle joint. She was treated with oral antibiotics. She rapidly deteriorated, became confused, pyrexial and developed a diffuse erythematous rash of her extremities. She was admitted to the Intensive Therapy Unit (ITU) and required intubation due to profound shock, cardio-respiratory failure and renal failure. Streptococcus pyogenes (b-haemolyticus Group A) was isolated from blood cultures and aspirate of the right ankle joint. A diagnosis of Streptococcal Toxic Shock Syndrome was made and she was started on high dose intravenous antibiotics.

**Figure 1:**
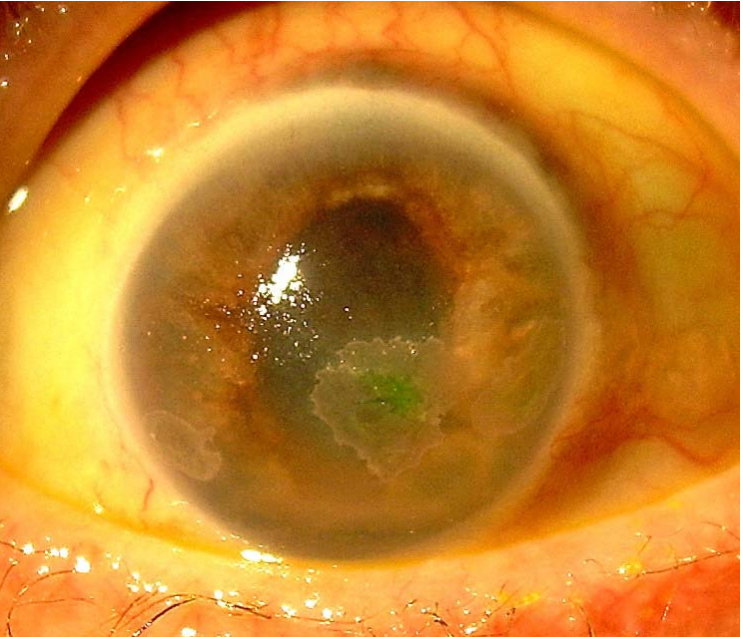
Colour photograph of the right eye showing the mid-dilated atrophic iris and the three inferior zones of keratopathy.

**Figure 2:**
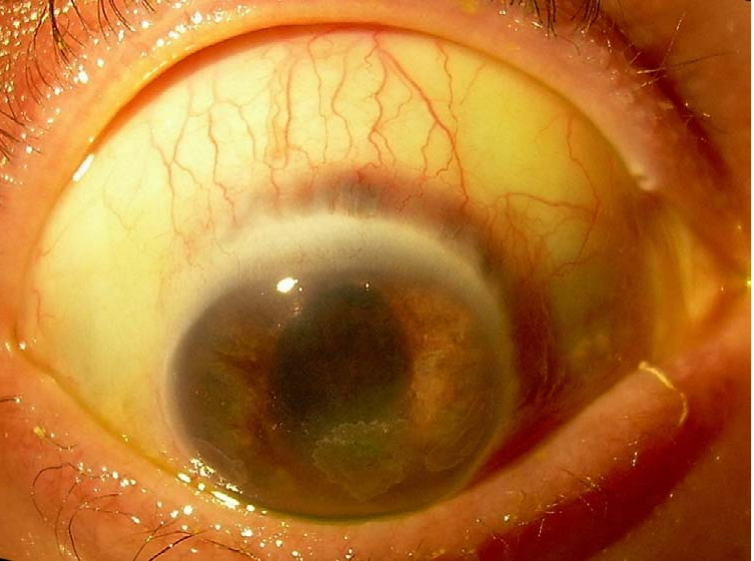
Colour photograph of the right eye indicating the initial thinning of the sclera at the superior limbus.

She was referred for ophthalmology assessment on day 36 of her admission to ITU as her right eye appeared injected and her right pupil was unresponsive to light. The visual acuity was reduced to hand movements (HM) on the right and was 6/6 on the left. The right eye was comfortable despite significant ciliary injection. She was examined by the bed side using a hand held slit lamp. On examination, the anterior chamber was deep and appeared quiet. The corneal surface appeared irregular with three distinct zones of sub-epithelial opacification. There was no corneal staining with 2% fluorescein. The right iris appeared atrophic and paler in colour. The pupil was fixed in mid-dilation with extensive posterior synechiae at 360 degrees (Fig 1). There were no transillumination defects or evidence of rubeosis iridis. There was no hypopyon. The intraocular pressure was elevated measuring 24 mmHg in the right eye. On indirect ophthalmoscopy the view of the fundus was clear with no evidence of inflammatory membranes in the vitreous. The retinal vessels appeared normal and there were no areas of intraretinal haemorrhage or pallor. The right optic disc was pale. The left eye was normal except for a small cotton wool spot above the left optic disc. A diagnosis of right optic atrophy in association with post-streptococcal uveitis was made and she was commenced on g maxidex hourly, g. cyclopentolate tds and g Timolol 0.25%. On day 48 the vision was perception of light (PL) in the right eye. Thinning of the superior sclera was noted and she was maintained on topical steroids (Fig 2). By this stage she had undergone extensive limb amputations including two below-knee amputations and bilateral amputations of all digits due to extensive vasculitic necrosis. The medical team were reluctant to treat her with oral steroids due to the risk of secondary infections in the healing wounds.

**Figure 3:**
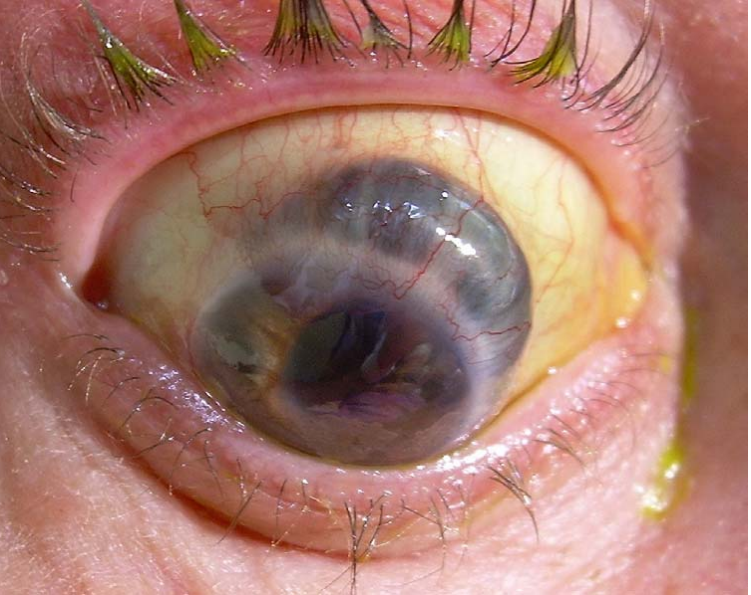
Colour photograph of the right eye showing the extensive thinning of the superior sclera and secondary anterior staphyloma formation.

By the fourth month a large anterior staphyloma had formed in the right eye between 11 and 4 o'clock positions (Fig 3). The eye remained comfortable and she was treated with g.maxidex qid and g timoptol 0.25% bid. At the last review, six months after presentation, the eye was comfortable. The vision remained PL on the right and 6/6 on the left. The intraocular pressure was 12 mmHg on g timoptol 0.25% b d on the right. Specific immunological tests conducted 5 months following the septicaemia showed normal levels of IgA, IgB, IgC, CD19/B cells and CD16/K cells. Pneumococcal antibodies were noted to be below the normal range.

## Discussion

Post-streptococcal uveitis is a rare clinical entity and to date it has been reported in 28 cases in the literature [1]. In most of these cases there was a history of anterior uveitis (more commonly non-granulomatous), with or without posterior segment involvement, following exposure to group A Streptococci and elevated Antistreptolysin O antigen titres [2]. The condition is often bilateral in presentation. Patients often present with a previous medical history that can be varied ranging from previous tonsillitis to rheumatic fever and toxic shock. In the former cases a high index of suspicion is required to make the association of bilateral anterior uveitis with possible exposure to group A streptococci.

The ocular features can be variable in this condition and can include bulbar conjunctival hyperaemia, anterior scleritis, scleral ectasia, keratic precipitates, vitreous opacities, choroiditis, retinal epithelial detachments and cystoid macular oedema [3].

It is suggested that the variability of presentation is a consequence of a host of cross-reacting antigens selectively targeting different parts of the uvea determined by the individual patient susceptibility. Other variables such as individual human leucocyte haplotypes, the virulence of the pathogen and the location deposition of circulating immune complexes may contribute to the diverse spectrum of ocular presentations [4].

Furthermore experimental studies have shown that intravitreal injection of streptococci or purified toxins initiate an inflammatory response in the iris and ciliary body with break-down of the blood ocular barrier. At very high doses of toxin, retinal and choroidal vessels appear to be compromised, thus representing a secondary site of breakdown resulting in anterior segment necrosis [5,6].

In this case the patient initially presented with septicaemia, shock and multi-organ failure. During her admission to ITU she developed an extensive necrotizing vasculitis involving all four distal extremities requiring distal amputations. We suspect that a similar immune complex-mediated mechanism led to involvement of the long and short posterior ciliary arteries producing an ischaemic involvement of the right optic nerve head, the iris, anterior sclera and the cornea. In addition, the presence of ciliary injection, extensive posterior synechiae and intraocular pressure elevation observed in the initial period suggest that post-streptococcal uveitis was also a factor in the right eye. We speculate that the presence of a transient single cotton wool spot in the left retina (lasting three weeks) was also indicative of a vasculitic process in the left eye that failed to progress, hence the marked asymmetry of disease between the two eyes. It is also interesting that in this case the clinical signs developed over a period of time as the patient initially presented with signs of anterior segment inflammation, keratitis and optic atrophy followed by the development of the necrotizing anterior scleritis. The latter developed gradually over a period of time.

We believe that the combination of an immune-mediated ischaemic vasculitis involving the peripheral extremities, anterior segment and a concurrent post-streptococcal uveitis has not been reported previously in a patient with Streptococcal Toxic Shock Syndrome.

This case illustrates the complex mechanisms at play in patients exposed to streptococcal antigens and the potential for severe ocular sequelae. It also highlights the importance of regular ocular review when these patients are in intensive therapy as they are bed-bound, often unconscious and therefore unable to report changes to their visual function. We recommend increased awareness of the potential risks of these patients developing severe ocular involvement.

## Abbreviations

DIC = Disseminated Intravascular Coagulation; HM = Hand movements; ICU = Intensive Care Unit; IOP = Intraocular Pressure; OD = Oculus dexter – right eye; OS = Oculus sinister – left eye; PL = Perception of light.

## Competing interests

The author(s) declare that they have no competing interests.

## Authors' contributions

KIP and ASI were involved in the conception and design, collection of data, analysis, literature research, writing and final approval of the article. PSA and AJS were involved in the critical revision and final approval of the article.

## Consent

Written informed consent was obtained from the patient for publication of this case report and accompanying images. A copy of the written consent is available for review by the Editor-in-Chief of this journal.
